# An Essay on the Sore-Mouth Peculiar to Nursing Females; Read before the New Albany Medical Society

**Published:** 1852-01

**Authors:** P. S. Shields

**Affiliations:** New Albany, Ia.


Art. V.—*An Essay on the Sore-Mouth peculiar to Nursing Females; read before the New Albany Medical Society.* By Dr. P. S. Shields, of New Albany, Ia.

In presenting a few thoughts on a disease peculiar to the nursing female, commonly called the "Nursing Sore Mouth," I do not propose or expect, to furnish a complete history of its symptoms, pathology, or treatment; but to direct the attention of the medical profession to a disease, which is endemic to our country—confined to certain regions or localities; is of somewhat frequent occurrence; and has not received from medical authors that degree of attention, which its frequency and importance demands.—The writer has searched in vain the various systems of practice, both European and American, and also many of the periodicals of the day, but can find nothing on this subject, except two papers, published in the American Journal of the Medical Sciences. The first is a communication from Dr. Backus, of Rochester, N. Y., to Dr. Bond, of Philadelphia, published in the January number for 1841; the other from the pen of Dr. E. Hale, of Boston, published in the medical communications of the Massachusetts Medical Society, and copied into the American Journal for April, 1842. It is surprising that the disease has not been noticed by our western journals, as I am persuaded it is one of very frequent occurrence in the valley of the Mississippi. My attention was first called to it as early as the year 1825 or '6, by our honored President, A. Clapp, M. D., when a pupil of his, and I have seen cases of it almost every year since commencing the practice of my profession. Dr. Backus has seen more or less of it in Rochester. for the last 25 years; and so has Dr. Hale, in Boston. And the editor of the Journal of Medical Sciences remarks, that Dr. Shanks, of Memphis, Tenn., says it is of frequent occurrence in that place.—But the editor further remarks, that he has never witnessed a case in Philadelphia; neither has the late Dr. Dewees, or any of the physicians of that city with whom he has conversed.

This disease is connected with, if not always produced by *Lactation;* I cannot say protracted, for it often makes its appearance as early as the first or second week, and generally between the fourth and eighth month; whilst many females continue to nurse for twenty or twenty-four months, without detriment to their health. Drs. Backus and Hale both say, that it attacks pregnant females towards the latter months of ultro-gestation. But I am persuaded they have confounded with it another form of sore-mouth, which is occasionally seen in the pregnant female. Dr. Hale says that it commences with a small red pimple upon the edge of the tongue, or inside of the lips, and soon forms a deep ulcer, with hard elevated edges, surrounded by an inflamed circle. Dr. Backus also speaks of it as occasionally commencing in this way. This form of sore-mouth is not confined to the pregnant female, but occasionally attacks the adult male, and children of both sexes; and is generally connected with derangement of the stomach and bowels. The writer has seen these ulcers, in one case, coexistent with the nursing sore-mouth, but regards it as merely incidental, as the nursing sore-mouth was almost cured, and the general health much improved, when they made their appearance.

The attack of sore-mouth is often very sudden—coming on in a few hours; but in most cases it is preceded by general lassitude, particularly a sinking weakness across the chest and epigastrium after nursing. This is followed by acid eructations, and burning sensation at the stomach. The first sensation in the mouth, is that of scalding, which is often intensely painful, and is accompanied by a profuse flow of a thin watery saliva, almost hot enough to excoriate the parts on which it may chance to flow. At the same time there is almost a complete destruction of the sense of taste. The profuse flow of saliva causes frequent spitting, which, connected with the great pain produced by the motion of the tongue, makes it often difficult for the patient to speak, and renders the articulation indistinct. Sometimes the appetite is good; at others there is little or none. The pain and irritation are sometimes so great that the reception of even the blandest articles of food causes great suffering to the patient.

This soreness of the mouth is confined principally to the under surface and edges of the tongue, extending back to its base, and sometimes to the palate; but seldom if ever to the point or dorsum of the tongue. The tongue is generally broader and thicker than natural, somewhat smoother, and of a light pinkish red appearance. Occasionally the papillæ are elevated, but the tongue is seldom if ever coated. There is often a slight abrasion, and in a few cases a portion of the tongue had the appearance as if the cuticle had been removed by a fly blister; but in no case has been seen a deep ulcer or any destruction of substance. These symptoms often continue for a week or ten days, causing great suffering; and then may partially or entirely disappear for a short time, and as suddenly return again in an aggravated form. Or they may gradually increase from week to week; accompanied by emaciation, and a long train of nervous symptoms, rendering the patient exceedingly wretched, until she is relieved by the weaning of the child, which generally quickly restores her to her wonted health and strength; or, if lactation be persevered in, and she is thus predisposed, phthisis or some other serious organic affection, may be thus developed. The bowels are generally rather slow, and the alvine evacuations of a dark green appearance. Dr. Hale says the soreness generally commences in the mouth, and then the inflammation extends to the mucous membrane of the esophagus, stomach, and bowels, causing diarrhœa; but according to my observation, the gastric symptoms precede the soreness of the mouth, and diarrhœa seldom appears; on the contrary the bowels being usually confined. The sore-mouth and diarrhœa sometimes alternate; the one curing or taking the place of the other.

There is always either a copious secretion of milk, or a preternatural richness in its quality; and the child is large and healthy. The mother usually says "that every thing she eats goes to milk." The pulse, at first, is but little altered in fulness or frequency. The skin is about its natural temperature. Dr. Hale says it is generally soft, and often attended by a profuse perspiration. This I have seldom witnessed.

There is much difference of opinion as to its pathology. That the local affection of the mouth is not the true seat of the disease may be inferred, becauss local remedies have little or no effect towards its removal. Besides, the gastric and constitutional symptoms precede those of the mouth. In one case under my care, the lady could always tell when an attack was coming on, from the burning sensation at the stomach, and the acid eructations, which came on an hour or two before the soreness of the mouth. If it were from inflammation of the stomach, we should have the gastric distress immediately on the reception of food, but those symptoms do not appear for an hour or two after eating. The disease also frequently comes on suddenly, and often disappears with equal suddenness, often without the use of any remedies.

It no doubt has its origin in debility, producing functional derangement of the stomach, and by either extension or sympathy, the mouth.

That lactation must produce this exhaustion of the vital powers, is evident from the large demand on the general system necessary to the unusually large secretion of milk. That it frequently attacks ladies possessing apparently robust constitutions, is no valid argument against the asthenic character of this disease, as we often see such persons have great intolerance of the loss of blood, or sink rapidly under any profuse discharge; whilst others possessing frail constitutions, or in feeble health, bear with impunity the same loss. There is also a great difference in the tenacity of life not to be ascertained by external conformation. When we contemplate the long continued and preternaturally large drain from the vital fluid of the nursing female, we need not be surprised that disease should follow. But why the mouth should be the part affected, in the nursing female, rather than some other part, we are not called upon to explain; neither is it necessary to the adoption of a correct treatment.

I am unable to give the post-mortem appearances, having never witnessed a fatal case.

*Treatment.*—If the foregoing views of its pathology be correct, it is manifest that the only certain means of cure, is to cease nursing before the development of any organic disease. According to my observation, a speedy restoration to health has always followed weaning. Drs. Backus and Hale make the same statement. But weaning the child may not always be necessary. By a proper course of treatment, it may sometimes be cured, or so much palliated, that she may continue to perform this important function without much suffering or danger to her life or future health. Neither can we always determine the propriety of weaning by the degree of soreness of the mouth; for many suffer severely from the mouth, while the gastric and constitutional symptoms are not at all urgent.

We should always try other means of cure, unless from the urgency of the symptoms weaning is rendered absolutely necessary to preserve the life of the patient. In my practice local applications have been of little or no service towards removing the disease. A solution of 15 grains of the nitrate of silver to ℥i of water, applied to the inflamed parts, by means of a camel's hair pencil, gives the most relief of any thing I have tried. A wash made of the acet. plumb. in water has occasionally proved beneficial. I once knew a lady greatly relieved by a wash made of a decoction of the blackberry-briar root with alum and white sugar. But our principal remedies must be directed to restoring the stomach and bowels to their healthy action, and giving tone to the general system.

I have generally commenced the treatment, by giving a gentle cathartic, such as blue mass at night, followed the next morning by rhubarb and calcined magnesia, to evacuate the intestinal canal, yet carefully avoiding active purging. The rhubarb and magnesia are then continued in smaller doses morning and evening, so as to keep the bowels in a soluble state, correct acidity of the stomach and give tone to the general system. We may give for the same purpose, a pill composed of equal parts of rhubarb, aloes and soap. Dr. Backus gives the following recipe:

℞ carb. ferri 55 grains, pulv. rhei and pulv. aloes, each 25 grains, pulv. ipecac and sapo., each 12 grains;—to be divided into 50 pills, two of which to be given morning, noon, and night; so as to keep the bowels quite loose. This, with the penciling of the tongue with the solution of nit. argent., constitutes his course of treatment.

As an antacid and tonic, the following has proved beneficial:

Carb. potass. ʒii, tinct. cinchon. ℥i, aq. destillat. ℥vi; mix and give ℥is. every four hours.

This I have found to set well on the stomach, and allay irritability of this organ when present. For the gastrodynia and pyrosis, the following pill morning, noon, and night, has been of much benefit:

Nit. bismuth. 2 grains, sulph. quin. 1 grain, sulph. morph. 1-12 grain.

In one case when these symptoms were severe, this prescription acted like a charm, completely removing them in a short time. When there is much derangement in the secretions of the liver as well as the stomach and bowels, and a dry skin, opium and calomel have proved valuable remedies. In one case, a pill at night gave great relief. In another, accompanied by diarrhea, the opium and hydrarg. cum creta proved beneficial. The sulph. quin. alone is recommended by Dr. Hale. He places his chief dependence upon tonics; among which he recommends the infusion of bark with lime water and the bottled porter, which I have also found beneficial. Various other tonics might be enumerated; but the above are those which have been used by the writer.

A resort to the pure country air contributes greatly to the recovery. The disease appears to be most frequently met with in our towns and cities situated on low grounds and large rivers, and seldom, if ever, visiting the high lands of the country.

As to the diet, the mild farinacious articles agree with the patient much better than animal food. Indeed, the smallest quantity of animal food has been known to bring on a severe attack of the burning sensation of the mouth. But we have to be governed by observation and experience in each case.

Those persons who have once had an attack appear more liable to its return.

I have attended a lady who has given birth to five children, and suffered from this disease while nursing every one. The first attack came on about the sixth month of lactation, while each subsequent one made its appearance earlier. The last came on about the third week of lactation. The acidity accompanied by the burning sensation at the stomach, and partial loss of taste appeared as early as the end of the first week.

New Albany, Ia., December, 1851.

